# Menstrual health and hygiene among Juang women: a particularly vulnerable tribal group in Odisha, India

**DOI:** 10.1186/s12978-023-01603-1

**Published:** 2023-03-30

**Authors:** Prasanna Kumar Mudi, Manas Ranjan Pradhan, Trupti Meher

**Affiliations:** 1 International Institute for Population Sciences, Deonar, Mumbai, 400088 India; 2 Department of Fertility and Social Demography, International Institute for Population Sciences, Mumbai, 400088 India; 3 International Institute for Population Sciences, Deonar, Mumbai, 400088 India

**Keywords:** Menstrual hygiene practices, Tribal women, India

## Abstract

**Background:**

Menstruation is a normal biological process that all women go through, yet it is shrouded in secrecy, taboos, and even stigma in many societies. Studies have shown that women from socially disadvantaged groups are more likely to have preventable reproductive health issues and have less understanding of hygienic menstrual practices. Therefore, this study aimed to provide an insight into the most sensitive issue of menstruation and menstrual hygiene practices among the women of the Juang tribe, recognized as one of the particularly vulnerable tribal groups (PVTG) in India.

**Methods:**

A cross-sectional study using a mixed-method approach was carried out among Juang women in Keonjhar district of Odisha, India. Quantitative data was gathered from 360 currently married women to assess practices associated with menstruation and its management. In addition, 15 focus group discussions and 15 in-depth interviews were conducted to explore the views of Juang women on menstrual hygiene practices, cultural beliefs, menstrual problems, and treatment-seeking behaviour. Inductive content analysis was used to analyse the qualitative data, while descriptive statistics and chi-squared tests were used to analyse quantitative data.

**Results:**

Most Juang women (85%) used old clothes as absorbents during menstruation. Distance from the market (36%), lack of awareness (31%), and high cost (15%) were cited as the contributing factors to the low level of sanitary napkin usage. Around 85% of women were restricted from participating in religious activities, and 94% avoided social gatherings. Seventy-one percent of the Juang women experienced menstrual problems, while only one-third of them sought treatment for their problems.

**Conclusion:**

Hygienic practices during menstruation are far from satisfactory among Juang women in Odisha, India. Menstrual problems are common, and the treatment sought is insufficient. There is a need for awareness generation on menstrual hygiene, the adverse effects of menstrual problems, and the provision of low-cost sanitary napkins among this disadvantaged, vulnerable tribal group.

## Introduction

Menstruation is a normal biological process that all women go through, yet it is shrouded in secrecy, taboos, and even stigma in many societies [1]. Menstruation integrates countless myths and mysteries, and not entering a religious place is one of the most widespread social and cultural norms and prohibitions about menstruation among women [2, 3]. During this time, women were considered impure and were often forbidden from entering places of worship or touching religious objects [4, 5]. In addition, menstruating women are also subjected to several other restrictions on household activities, such as entering the kitchen, cooking, touching pickles, and sharing a meal [6, 7]. Menstruation is not only a taboo topic for public discussion in India but is also rarely discussed in private. This often leads to misconceptions and a lack of preparedness among women [5]. Inadequate or erroneous knowledge about menstruation is often the source of unwarranted constraints on the everyday routine activities of menstruating females, resulting in various psychological problems [8]. Although the situation is changing among educated and urban women, such behaviours are still prevalent in rural areas [9].

Maintaining menstrual hygiene is of utmost importance for menstruating girls and women. The notion of maintaining the cleanliness of the body during menstruation is known as menstrual hygiene. It necessitates simple amenities such as hygienic absorbent material, water, soap, and toilet facilities with privacy [10]. The selection and effective usage of menstrual absorbents is a crucial aspect of menstrual hygiene management (MHM). A review by Chandra-Mouli et al. [11] suggests that adults' degree of menstrual supervision may contribute to differences in fundamental MHM habits such as absorbent usage, frequency of change, and daily bathing routines. The World Health Organization (WHO) states that around 1.7 billion people worldwide lack access to basic sanitation [12]. In addition, due to a lack of knowledge and high cost, women also use old clothes and other unhygienic materials as menstrual absorbents. This lack of knowledge and poor personal hygiene during menstruation has been linked to major health problems such as genital and urinary tract infections [6, 13, 14]. A hospital-based cross-sectional study found a strong association between poor menstrual hygiene practices and a higher prevalence of reproductive tract infections (RTIs) among women in Odisha [15]. These repercussions impair a woman's capacity to support herself and her family and ultimately impact a country's economy. Therefore, a better understanding of menstrual hygiene management is crucial for women's health and dignity. Despite the fact that menstrual hygiene is an important issue that has long been kept hidden, there is a long-standing need to confront it openly. Unaddressed menstrual hygiene is considered to obstruct the attainment of several Sustainable Development Goals (SDGs) since it is linked to gender equality and female empowerment through its direct impact on women's reproductive health, education, and work engagement.

In India, the social group plays a vital role in determining access to social and economic resources [16, 17]. Socially backward groups, such as scheduled caste (SC) and scheduled tribe (ST), often fall behind in most socioeconomic development and health indices, including health care knowledge and usage [18]. There is a dearth of information among socially deprived communities regarding the various measures required to preserve the health and dignity of women during menstruation. Additionally, several studies have shown that women from these socially disadvantaged groups are more likely to have preventable reproductive health issues and have less understanding of hygienic menstrual practices [19–21]. A previous study among adolescent girls in a tribal district of Odisha has reported that more than one-third of girls use unsanitary materials as menstrual absorbents [22]. Despite the discomfort caused by using cloth, it continues to be their preferred method due to its ease of availability, low cost, and reusability. Another study reveals that the knowledge about menstruation is poor among Munda tribal adolescent girls in Odisha, and practices included various myths and misconceptions [23]. The recent National Family Health Survey (2019–21) also found that 59% of the tribal women aged 15–24 use cloth as menstrual absorbents compared with 25% of their counterparts from the general caste [24].

Considering the above scenario, the current study about menstrual health and practices was conducted on a sample of women from the Juang tribe, recognized as a Particularly Vulnerable Tribal Group (PVTG) by the Government of India. Odisha is home to 13 of the 75 PVTGs on the list. They are primarily homogeneous, with a limited population and a geographically secluded location. A pre-agricultural way of life-based on hunting and gathering, relatively stagnant population growth, primitive economy, and extremely low literacy levels compared to other tribal groups are all characteristics of such groups [24, 25]. Several anthropological studies have revealed the Juangs' deep attachment to traditional rituals and health beliefs, yet, scientific research on reproductive health in general, and menstrual hygiene practices in particular, is scarce. Therefore, this study aimed to provide insight into the issue of menstruation and the status of hygiene and practices regarding menstruation among one of these primitive tribal groups in Odisha, India.

## Methods

This cross-sectional study using a mixed-method approach was conducted among the Juang tribe in the Keonjhar district of Odisha, India. The mixed-method approach was explanatory mixed-method, i.e., the authors first collected the quantitative data, and after preliminary analysis of quantitative information, the qualitative data was gathered. According to the 2011 census, the population of the Juang community was around 47,095, with a majority of them living in rural areas, particularly in the districts of Keonjhar and Dhenkanal in Odisha [26]. The community is mainly divided into two parts, namely the hill Juangs, those confined to the hill ranges of Keonjhar and Pallahara, and the plain Juangs, those distributed among the plains of Dhenkanal and Keonjhar districts of Odisha. The hill Juangs are still in a primitive stage, relying primarily on shifting cultivation, whereas the plains Juangs have adopted settled agriculture [27]. They are also known for their adherence to traditional customs and health beliefs. The Juang tribes belong to the Munda ethnic group and speak the Juang language, which is accepted as a branch of the more exceptional Austroasiatic Language Family. A past study also found that Juangs continue to exhibit a primitive level of sociodemographic status, low literacy rate, very poor maternal and child healthcare practices, and a high prevalence of undernutrition [27].

The study adopted a three-stage sampling design to choose respondents, i.e., currently married women aged 15–49. In the first stage, three tehsils, i.e., administrative divisions in a district (Banspal, Telkoi, and Harichandanpur), were randomly selected from the 13 tehsils having villages with the Juang tribe in the Keonjhar district of Odisha, India. Secondly, after listing all villages with at least 50 Juang households in these tehsils, six villages (2 each from each tehsil) were randomly selected. During household mapping and listing, the availability of married women aged 15–49 was asked to identify the households having eligible respondents in these six villages. All the listed eligible women were interviewed (n = 360). The sample size was decided considering (a) the concentration of the respondents in the district, (b) the minimum sample size for any meaningful analysis, and (c) available resources such as time and cost. The larger study also interviewed the husband of the eligible women in every third randomly selected household. In addition, 15 in-depth interviews (IDI) of Juang women and 15 focus group discussions (FGD) with community members were conducted to learn more about their lifestyles, gender norms around reproductive health, and male engagement in reproductive health. The respondents for the qualitative interviews were chosen purposively. The first author collected the quantitative data through a pretested interview schedule by visiting the households between 2020 and 2021 with an in-between pause due to the COVID-19-induced lockdown. A pre-tested checklist was further used to gather the qualitative data from the study area. The present analysis is based on the quantitative data of 360 women and qualitative interviews.

### Analysis

**Fig. 1:**
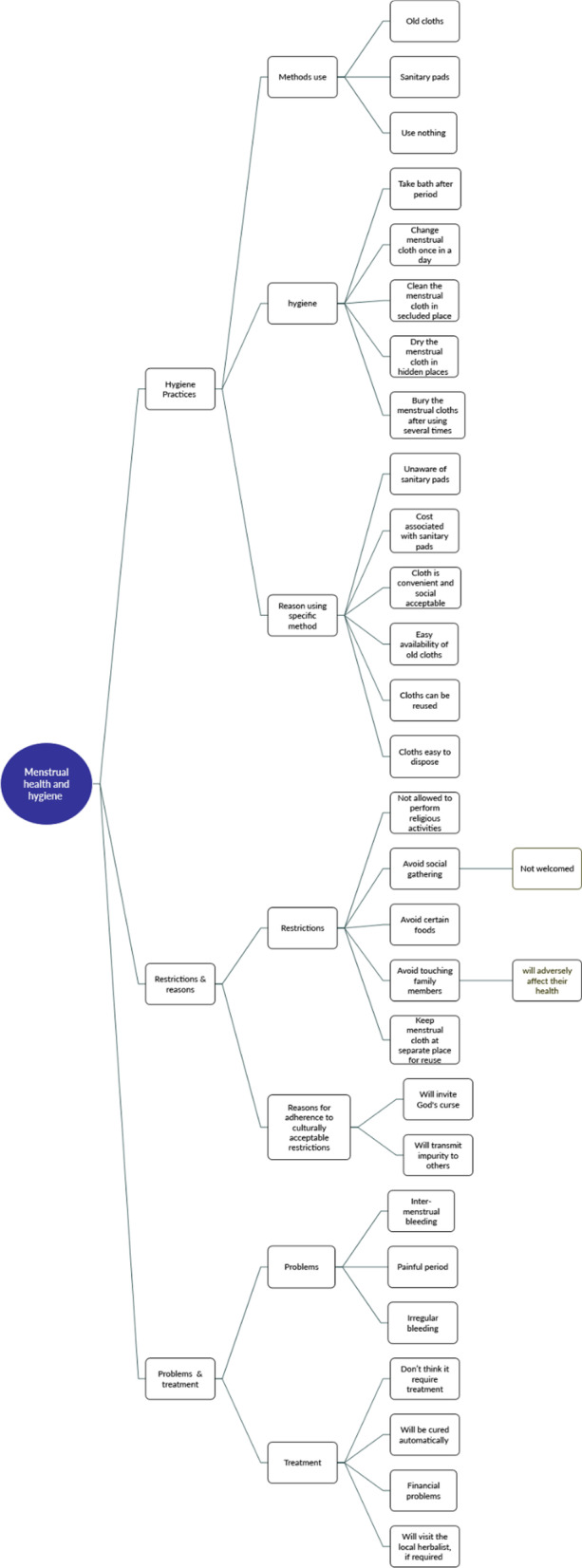
Mind map of menstrual health and hygiene

To begin, quantitative data were entered using the CSpro. 4.0 software and analyzed using STATA (V16). A descriptive analysis was carried out to see the distribution of the Juang women by several socioeconomic and demographic characteristics. Bivariate analysis was conducted to check the independent association of various socioeconomic and demographic characteristics with absorbents used to prevent menstrual blood from being evident and the prevalence of any menstrual problems. Content analysis of the qualitative data was carried out using NVivo (V12). Anonymized direct quotes from the respondents were given to supplement the quantitative findings wherever applicable. The study further presents the mind map describing the codes, categories, and themes emerging for the qualitative data analysis (Fig. 1).

### Ethical concern

The study was conducted among the Juang tribe, a PVTG primarily found in Odisha, India. At first, ethical approval was obtained from the student research ethics committee of the institute affiliated with the authors. The first author briefed the study objectives to the appropriate authorities/chiefs of the villages chosen for the study and secured their approval. The community and individuals were told of the study's purpose before the survey, and they were assured that their information would be used purely for academic research. Additionally, individual respondents' agreements were also sought before the interview, with the assurance that they might withdraw at any time. The study only interviewed participants who gave their assent willingly.

## Results

### Socio-demographic profile of the Juang women

**Fig. 2:**
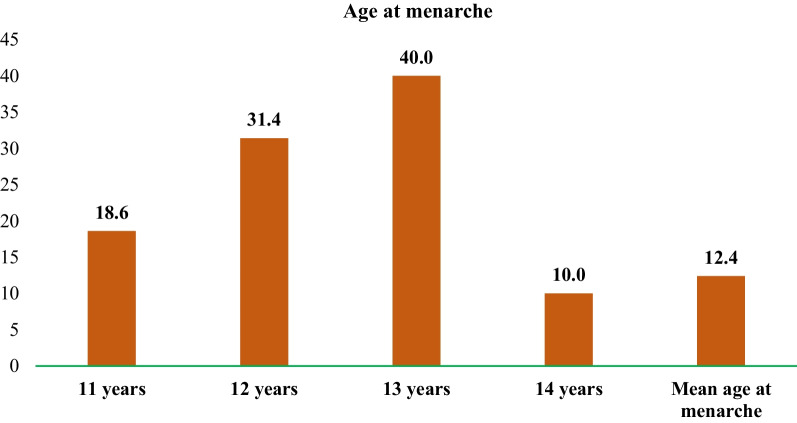
Distribution of age at menarche among Juang women

The mean age of the 360 women interviewed in the study was 26 years (Table 1). Of the total women, 44% were aged 15–24 years, (31%) were aged 25–29 years, and the rest were aged 30 and above. Two-thirds of the women were illiterate, and about three-fifths were not working. Most of the women (89%) reside in nuclear families. Nearly three-fifths of the women consumed tobacco and alcohol and were not exposed to any mass media (63%). Two-thirds of the women were from households without any toilet facilities. Of the women, 60% had Kachha houses, and 20% had Semi-pucca or Pucca houses. The mean age at menarche for the studied women was 12.4 years. Nineteen percent of the women reached menarche at the age of eleven, 31% at the age of twelve, 40% at the age of thirteen, and 10% at the age of fourteen (Fig. 2).

**Table 1:** Socio-economic and demographic profile of Juang women in Odisha, India

Background characteristics	Total (N = 360)	
	%	N
*Age group*		
15–24 years	44.4	160
25–29 years	31.1	112
≥ 30 years	24.4	88
Mean age of surveyed women	26.0	
*Education*		
Illiterate	66.4	239
Literate	33.4	121
*Working status*		
Not working	59.2	213
Working	40.8	147
*Type of family*		
Nuclear	88.6	319
Joint	11.4	41
*Usage of tobacco*		
No	41.9	151
Yes	58.1	209
*Consumption of alcohol*		
No	40.6	146
Yes	59.4	214
*Media exposure*		
No exposure	62.6	225
Any exposure	37.4	135
*Type of toilet facility*		
Pit latrine	33.6	121
Use open space/field	66.4	239
*Type of house*		
Kachha	60.0	216
Semi-pucca	19.7	71
Pucca	20.3	73

### Hygiene practices during menstruation

Most Juang women (85%) used old clothes as absorbents during the menstrual period (Table 2). Additionally, 9% used sanitary napkins/locally prepared napkins, and 6% did not use anything as absorbent during their menstrual cycle. Eighty-two percent of the women cleaned their external genitalia with only water and 18% with water and soap during menstruation. Of those using cloth absorbents, 53% changed them once, while 41% changed them twice and 6% thrice a day. Most women (81%) cleaned their menstrual clothes with only water. More than half of the women (56%) dried their menstrual clothes inside the house but in a hidden place; 32% dried them inside the house but in an open space, and the remaining dried their menstrual clothes outside the house in the sunlight. Moreover, 82 percent of women stored their menstrual clothes in hidden places, whereas 18 percent usually kept them with their regular clothes.

**Table 2:** Distribution of Juang women based on their hygiene practices during menstruation

Hygiene practices	%	N
*Type of absorbent used*		
Sanitary napkin	9.2	33
Cloths	85.3	307
Nothing	5.6	20
*Frequency of changing menstrual cloth*		
1 time a day	52.8	162
2 times a day	41.4	127
3 times a day	5.9	18
*Cleaning of menstrual cloth*		
With water	81.8	252
With soap and water	16.6	51
Don’t clean	1.6	5
*Place of drying menstrual cloth*		
Inside the house but in hiding	56.4	173
Inside the house but in open	31.9	98
Outside and in sunlight	11.7	36
*Place of keeping reused menstrual cloth*		
Normal like other cloths	18.2	56
At hidden place	81.8	251
*Cleaning of external genitalia*		
With water	81.9	295
With soap and water	18.1	65

**Fig. 3:**
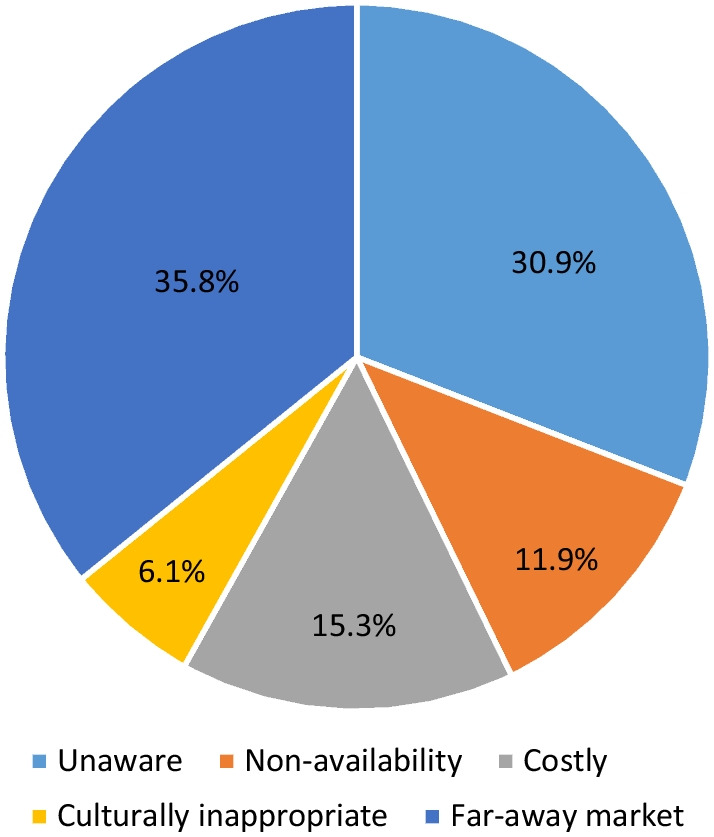
Reasons for not using sanitary napkin among Juang women

Table 3 presents the types of absorbents used during menstruation by the women's background characteristics. With increasing age, the use of cloth as absorbent decreases, and sanitary napkins/locally prepared napkins increase. For instance, eight percent of the women aged 15–24 used sanitary/locally prepared sanitary napkins, while the corresponding figure was 10% among women aged 25–29 years and 11% among women aged 30 and above. Nineteen percent of those literate women, compared with 4% of illiterate women, used sanitary/locally prepared sanitary napkins. Thirteen percent of those working women used sanitary/locally prepared sanitary napkins, which is only 7% among those not-working. Of the women from joint families, 12% used sanitary/locally prepared napkins, compared with 9% from nuclear families. A higher percentage of women (16%) not consuming alcohol and tobacco used sanitary/locally prepared sanitary napkins than those consuming them (4%). Sixteen percent of the women with any media exposure used sanitary/locally prepared sanitary napkins, while 5% among the women without any exposure. Fifteen percent of the women from households with a pucca house used sanitary/locally prepared napkins. The corresponding figures were 13% among those from semi-pucca households and 6% among those with kaccha houses. During menstruation, 8% of illiterate women use nothing. Distance to the market where one can get the sanitary napkin/locally prepared sanitary napkins (36%), followed by unawareness of sanitary napkins (31%), the cost associated with sanitary napkins (15%), non-availability (12%), and cultural inappropriateness (6%) were the reasons for non-use of sanitary napkins among Juang women (Fig. 3).

**Table 3:** Type of absorbent used by Juang women during menstruation by background characteristics

Background characteristics	Type of absorbent (%)			p values
	Sanitary napkin	Clothes	Nothing	
*Age group*				p = 0.137
15–24 years	7.5	89.4	3.1	
25–29 years	9.8	84.8	5.4	
≥ 30 years	11.4	78.4	10.2	
*Education*				p < 0.001
Illiterate	19.0	80.2	0.8	
Literate	4.2	87.9	8.0	
*Working status*				p = 0.072
Not working	6.6	88.7	4.7	
Working	12.9	80.3	6.8	
*Type of family*				p < 0.001
Nuclear	7.6	86.7	5.7	
Joint	20.0	75.6	4.4	
*Usage of tobacco*				p < 0.001
No	3.8	90.4	5.7	
Yes	16.6	78.2	5.3	
*Consumption of alcohol*				p < 0.001
No	4.2	87.4	8.4	
Yes	16.4	82.2	1.4	
*Media exposure*				p = 0.001
No exposure	4.9	88.4	6.7	
Any exposure	16.4	80.6	3.0	
*Type of toilet facility*				p < 0.001
Pit latrine	18.2	76.0	5.8	
Open defecation	4.6	90.0	5.4	
*Type of house*				p = 0.053
Kachha	6.0	87.0	6.9	
Semi-pucca	12.7	81.7	5.6	
Pucca	15.1	83.6	1.4

Qualitative data also revealed that the cost associated with sanitary napkins was a major barrier to their usage. In the words of an in-depth interview participant, "We cannot purchase sanitary napkins. We do not have money. Whatever money we have is for our daily livelihood. Cloths are well-known to be used, and it is easy to use three to four times. Sanitary napkins are only used once. (IDI-P3)". The cloth was convenient for the women and could be reused without additional cost.. Furthermore, the FGDs found that women washed their menstrual cloth privately, usually on the river bank (called ghat), where people do not bathe. Moreover, they dried these menstrual cloths in the backside of their huts/houses, away from the eyes of other family members. Washing menstrual clothes in the usual bath places and drying them in open spaces are strictly avoided because it is believed to be impure and affect the health of people coming in contact. Moreover, some women feel it very embarrassing to dry it along with other usual cloths. To cite one woman FGD participant: “We usually use kaffin (a piece of old cloth) during menses (menstruation). Wash it the next day for next time use. We wash our menstrual cloths in the river or pond in separate ghats where no one bathes, and we dry them on the backsides of house roofs so that no one notices." (FGD-1).

Some IDI participants also drew attention to the general negative attitude towards clothes used during menstruation and storage. One IDI respondent viewed, “I keep my menstrual clothes in a different spot (hidden), where other clothes would not touch, since if they do, it will become impure.” (IDI-P5) Another respondent said: “Once I put my menstrual clothes in the same place as other clothes, my husband scolded me badly that day and did not speak to me for a week.” (IDI-P6) Moreover, after three to four uses, women usually bury the used 'kaffin' under the mud of a nearby pond or riverbed so that it does not come into contact with another person. This is due to the belief that these impure clothes will adversely affect people's health if contacted, and to avoid embarrassment if it comes to the notice of other people. One IDI participant said, "I feel shy and wait for an appropriate time when nobody will be in the ghat so that I can bury the kaffin secretly". (IDI-P15) Another woman viewed, "I usually wash my menstrual clothes in the evening so that nobody can see it." (IDI-P11).

### Restrictions practiced during menstruation among Juang women

**Fig. 4:**
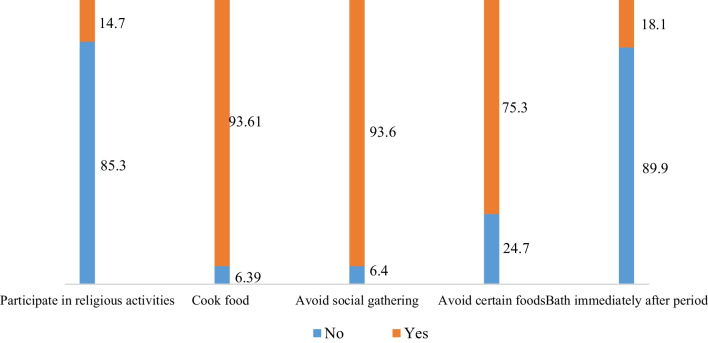
Restrictions practiced during menstruation among Juang women

Juang women described a variety of socio-cultural taboos and restrictions imposed on them during their menstruation (Fig. 4). Around 85 percent of women were restricted from participating in any religious activities. Nearly three-quarters of the sampled women (75%) avoid certain food items, and 94% avoid social gatherings. Less than one-fifth (18%) of women reported taking baths immediately after their periods. Focus group discussions also revealed several restrictions culturally enforced upon menstruating women. Some women perceive visiting religious places during menstruation will attract God’s curse as it is associated with the impurity of women. In the words of an illiterate working woman, “We are not permitted to visit a holy place during our menstruation. We also avoid social events and celebrations in our hamlet because doing so would contribute to the family's impureness, and the gods will curse the family.” (FGD-2). Another IDI participant perceived that even touching other family members and strangers during menstruation is not recommended, as the woman will transmit the impurity. In her words, “we do not touch any family members or strangers since it feels like it will pass the impurity to them.” (IDI-P9).

### Menstrual problems and treatment-seeking behaviour

Table 4 presents the prevalence of menstrual problems by background characteristics of Juang women. More than two-thirds (71%) of the Juang women experienced menstrual problems during the last six months preceding the survey (Table 5). Of the women aged 15–24, 66% had menstrual problems. The corresponding figures were 72% and 76%, respectively, among those aged 25–29 years and 30 + years. Most literate women (97%) reported experiencing menstrual problems than their illiterate counterparts (57%). Working women (76%), those from joint families (85%), those who used tobacco (85%), drank alcohol (94%), and those who were exposed to mass media (81%) had higher rates of menstrual problems than their counterparts. Eighty-nine percent of the women from households with pit latrines had menstrual problems, which was 61% among women without any toilet. Three-fourths of the women residing in Pucca houses had menstrual problems compared with 69% among women residing in Kachha houses.

**Table 4:** Menstrual problems during last six months preceding the survey among Juang women by background characteristics

Background characteristics	Menstrual problems (%)	p values
*Age group*		p = 0.233
15–24 years	66.3	
25–29 years	72.3	
≥ 30 years	76.1	
*Education*		p < 0.001
Illiterate	56.9	
Literate	97.5	
*Working status*		p < 0.001
Not working	67.1	
Working	75.5	
*Type of family*		p = 0.001
Nuclear	68.7	
Joint family	85.4	
*Usage of tobacco*		p < 0.001
No	60.3	
Yes	84.8	
*Consumption of alcohol*		p < 0.001
No	54.7	
Yes	93.8	
*Media exposure*		p < 0.001
No	64.3	
Yes	81.3	
*Type of toilet facility*		p < 0.001
Pit latrine	89.3	
Open defecation	61.1	
*Type of house*		p < 0.001
Kachha	69.0	
Semi-pucca	70.4	
Pucca	75.3

**Table 5:** Menstrual problems and treatment seeking behaviour among Juang women

Background characteristics	%	N
*Any menstrual problem*		
No	28.6	103
Yes	71.4	257
*Types of menstrual problem*		
Painful periods	29.2	75
Inter-menstrual bleeding/others	44.0	113
Scanty bleeding	26.9	69
*Sought treatment for menstrual problem*		
Yes	32.7	84
No	67.3	173
*Reason for not seeking any treatment*		
Cured automatically	39.9	69
Felt treatment not required	12.1	21
Financial problem	15.0	26
No nearby health provider	16.8	29
No time to go for treatment	16.2	28

Of the women with any menstrual problems, 44% reported inter-menstrual bleeding, 29% reported painful periods, and 27% reported experiencing scanty bleeding (Table 5). Qualitative interviews also found that many women experience irregular periods which is perceived as a normal phenomenon. To quote an IDI participant, "Sometimes I miss my monthly period, but it resumes next month" (IDI-P14). Only one-third of the women with any menstrual problems sought treatment. Cured automatically (40%), felt the treatment was not required (12%), financial problems (15%), no nearby health provider (17%), and no time to go for treatment (16%) were cited as the reasons for not seeking treatment for any menstrual problem. Qualitative data reveal that besides poor economic conditions, lack of awareness about menstrual problems and their consequences was the major barrier to treatment-seeking for any menstrual health problem. In the words of an IDI participant, "I was told it (painful period) is normal and hence does not require medical treatment.” (IDI-P10) Nevertheless, the local herbalist was the preferred choice for any reproductive health-related problems among women in the community. To quote a FGD participant, “We usually consult the Vaidya in the adjacent village for menstrual and other gynaecological problems.” (FGD-7).

## Discussion

The study found poor menstrual health and hygiene practices among Juang women. Lack of knowledge, availability, and financial constraints are barriers to using sanitary napkins. Cultural acceptability and easy availability, convenience to use, reuse, and disposal influence the higher use of cloths as menstrual absorbents. Despite most women experiencing menstrual problems, the treatment sought is inadequate. Furthermore, Juang women reported a variety of socio-cultural taboos and restrictions imposed on them concerning menstruation.

The mean age at menarche among Juang women was 12.4 years, and the findings conform to earlier studies by Paria et al. [28] and Pandey and Pradhan [29]. The study revealed that most Juang women used old clothes as menstrual absorbentsJuang. This is consistent with the findings of earlier research that found a significant preference for old clothes as menstruation absorbents over sanitary pads [6, 8, 30, 31]. Furthermore, according to a study conducted in Rajasthan, three-fourths of the girls reported using old clothes during menstruation, whereas only one-fifth reported using sanitary napkins [32]. Lack of awareness was one of the significant reasons for the low usage of sanitary napkins among Juang women. Moreover, lack of access to sanitary napkins due to various factors such as distance from the market, high cost, and non-availability was also reported as contributing to the low sanitary napkin usage. Good hygienic practices, such as using sanitary napkins and adequately cleaning the genital area, are essential during menstruation. The type of absorbent used is of primary concern, as reusing the material might lead to infection if not properly cleaned and stored [30]. A hygienic menstrual absorbent enables women to manage menstruation safely, comfortably, and effectively [10].

Water was the most common agent used by most Juang women for cleaning external genitalia during menstruation. The current study also observed that in a sizable number of Juang women who used cloths as menstrual absorbent, the frequency of changing absorbent was only once per day, and a majority cleaned the menstrual cloths only with water. The drying of menstrual cloth in sunlight and its storage in a hygienic place for reuse is equally essential for good hygiene, which only a tiny percentage of Juang women were following, often due to the taboo associated with menstruation. A past study also found that taboos and embarrassment associated with menstruation make it difficult for women to wash and dry menstrual absorbents in a sanitary manner [33]. Such unhygienic and improper menstrual practices can increase the risk of reproductive tract infection and other health hazards [34, 35]. Previous research has also raised concerns about inadequate washing and drying of menstrual absorbents [5, 6, 21].

This study also revealed that Juang women were restricted from participating in any religious activities during menstruation. They also avoid certain food items and social gatherings during periods. Previous studies have also reported similar restrictions women, practice during menstruation [9, 36, 37]. The inadequate understanding of menstruation as a biological process and adherence to myths and misconceptions such as impurity often compel women to follow these practices silently. The cultural belief that non-adherence to existing practices and attending religious activities and social gatherings may result in an adverse outcome for the women and family further compels Juang women to follow socio-culturally sanctioned restrictions during menstruation.

JuangThe study found a majority of Juang women reported having menstrual problems. However, only around one-third of them sought treatment for their problems. This finding is concurrent with the study by Mathiyalagen et al. [38] and Kulkarni and Durge et al. [39]. Lack of awareness about menstrual problems and their consequences, assuming symptoms normal, besides economic constraints, were the major barriers to treatment-seeking for any menstrual health problem among Juang women. A past study also found similar barriers to treatment seeking for Menstrual problems [40]. Previous small-scale studies have documented a considerable lack of knowledge of menstruation and its management among tribal women in different parts of India [20, 21]. Evidence suggests menstrual disorders affect the quality of life of women [41, 42].

### Methodological considerations and limitations

It is a mixed-method cross-sectional study that addresses the problem, hygienic behaviors, and cultural beliefs that Juang women encounter during menstruation. The findings from the quantitative survey and the qualitative interviews were triangulated to understand the issue more comprehensively. Our findings provide some baseline information on prospective differences that might be investigated further in future studies to enable more context-specific menstrual hygiene practices and treatments for menstrual problems. However, large-scale statistics do not address specific groups or communities' issues, such as PVTGs. The self-reported nature of the data can be subjected to reporting bias. In addition, there would probably be some information bias as the study was conducted among tribal women with a meager literacy rate. Future clinical studies can reveal the exact prevalence of menstrual problems among Juang Women. Our research focuses on menstrual hygiene practices and will augment existing programmes and policies to help the Juang tribes and other PVTGs improve their reproductive health. Despite these limitations, this is the first study to our knowledge that investigates menstrual hygiene practices among the Juang tribe, highlighting the menstrual hygiene problems and treatment-seeking behaviour. A strong blend of qualitative and quantitative tools to study and quantify various parts of this complicated topic is also one of the strengths of our work.

Gathering the required data from the study population was a challenge in itself. The villages selected for the fieldwork were located in remote areas, especially in the foothills surrounded by forests, which was the first challenge the researcher had to cross to reach the study population. Securing permission from the village head for the study and ensuring consent from them as well as the respondent was possible after repeated visits to the study area and providing details of the study to all concerned. As the researcher was familiar with the local language, a variant of the Odia (the state’s official language), the concerns about communication with this primitive tribe could be addressed. Most of the interviews were conducted early morning or late afternoon, given the respondents' availability and work schedule. Although the researcher had a prior understanding of tribal culture and lifestyle, efforts were made so that researcher reflectivity did not affect the entire research process, including gathering, analyzing, and presenting the results.

## Conclusion

Hygienic practices during menstruation are far from satisfactory among Juang women in Odisha, India. Most of them cannot use sanitary napkins due to a lack of knowledge, availability, and financial constraints. Menstrual problems are common among Juang women. However, the treatment sought is insufficient. These findings suggest the need for awareness generation on menstrual hygiene and the adverse effects of menstrual problems on the health and welfare of this disadvantaged, vulnerable tribal group. Moreover, efforts to provide free or low-cost sanitary napkins to Juang women at doorsteps through grassroots-level health workers may enhance its use. Socio-cultural norms negatively influence the Juang women's menstrual health and hygiene practices. Hence, a customized program involving multiple stakeholders to create a conducive environment that addresses taboos/myths/misconceptions surrounding menstruation and the menstrual health of the Juang women seems pertinent.

Despite the government’s efforts to distribute affordable sanitary napkins through programmes like Rashtriya Kishor Swasthya Karayakram (RKSK), the outcome appears to be far from satisfactory among primitive tribal women. Therefore, more effort should be made to educate these hard-to-reach PVTGs, about the need for MHM and offer them free sanitary napkins to increase their usage. The Juang Development Agency (JDA) and Integrated Tribal Development Agency (ITDA), which are government initiatives entrusted with tribal development along with other local Non-governmental organizations (NGO) may use these findings to frame program to ensure improved menstrual health of Juang women and similar PVTGs. Improved reproductive health of PVTGs will help to achieve Goals 3 and 5 of the SDGs.

## Data Availability

The datasets generated and/or analysed during the current study are not publicly available due to the reason that it was gathered for the doctoral research of the first and corresponding author by the first author, but are available from the corresponding author on reasonable request.
